# Thrombopoietin induces production of nucleated thrombocytes from liver cells in *Xenopus laevis*

**DOI:** 10.1038/srep18519

**Published:** 2015-12-21

**Authors:** Yuta Tanizaki, Megumi Ichisugi, Miyako Obuchi-Shimoji, Takako Ishida-Iwata, Ayaka Tahara-Mogi, Mizue Meguro-Ishikawa, Takashi Kato

**Affiliations:** 1 Department of Biology, Faculty of Education and Integrated Arts and Sciences, Waseda University, 2-2 Wakamatsu, Shinjuku, Tokyo, 162-8480, Japan; 2 Integrative Bioscience and Biomedical Engineering, Graduate School of Advanced Science and Engineering, Waseda University, 2-2 Wakamatsu, Shinjuku, Tokyo 162-8480, Japan

## Abstract

The development of mammalian megakaryocytes (MKs) and platelets, which are thought to be absent in non-mammals, is primarily regulated by the thrombopoietin (TPO)/Mpl system. Although non-mammals possess nucleated thrombocytes instead of platelets, the features of nucleated thrombocyte progenitors remain to be clarified. Here, we provide the general features of TPO using *Xenopus laevis* TPO (*xl*TPO). Hepatic and splenic cells were cultured in liquid suspension with recombinant *xl*TPO. These cells differentiated into large, round, polyploid CD41-expressing cells and were classified as *X*. *laevis* MKs, comparable to mammalian MKs. The subsequent culture of MKs after removal of *xl*TPO produced mature, spindle-shaped thrombocytes that were activated by thrombin, thereby altering their morphology. *Xl*TPO induced MKs in cultured hepatic cells for at least three weeks; however, this was not observed in splenic cells; this result demonstrates the origin of early haematopoietic progenitors in the liver rather than the spleen. Additionally, *xl*TPO enhanced viability of peripheral thrombocytes, indicating the *xl*TPO-Mpl pathway stimulates anti-apoptotic in peripheral thrombocytes. The development of thrombocytes from MKs via the TPO-Mpl system in *X. laevis* plays a crucial role in their development from MKs, comparable to mammalian thrombopoiesis. Thus, our results offer insight into the cellular evolution of platelets/MKs in vertebrates. (200/200).

Platelets are generated from the cytoplasm of polyploid megakaryocytes (MKs). In humans, MKs differentiate from haematopoietic stem cells (HSCs) and constitute only a small fraction of bone marrow cells (0.1%–0.5%)^1^. MKs are unique cells that undergo DNA replication, giving rise to polyploid cells that undergo proplatelet formation^2^. The proliferation and maturation of MKs by thrombopoietin (TPO), a ligand for the receptor encoded by the *c-mpl* proto-oncogene (Mpl)^3^,^4^,^5^, has been well characterized. TPO has been independently identified and purified from different species in mammals^6^,^7^. In contrast, the origin and development of circulating nucleated thrombocytes in most non-mammalian vertebrates, including fish^8^,^9^,^10^, amphibians^11^, reptiles^12^ and aves^13^, remain unknown^14^,^15^. The evolutionary advantage of deriving platelets from MKs has been previously discussed^16^. Circulating thrombocytes mediate haemostasis and blood coagulation, and result in the activation and cytoskeletal changes of non-mammalian nucleated thrombocytes, similar to those of platelets^17^. In zebrafish, thrombin activates nucleated thrombocytes produced by TPO stimulation^18^. Nevertheless, it is not clear whether polyploid MKs are the precursors of mature nucleate thrombocytes.

In humans, HSCs develop into committed multipotent progenitors, which in turn differentiate to produce lymphocyte progenitors, granulocyte/monocyte progenitors, and MK/erythroid progenitors (MEPs). MEPs committed to the formation of erythroid and megakaryocytic progeny then produce mature erythrocytes or platelets^19^. Although TPO is one of the most important inducers of MK maturation, high concentrations of TPO inhibit proplatelet formation *in vitro*^20^. Recently, Nishimura *et al.* reported that the IL-1α also stimulates platelet production in response to acute platelet needs^21^. Newly released peripheral platelets exhibit bipolar morphology of round cells and multi-bodied proplatelets^22^. Proplatelet formation and platelet release are accelerated by shear forces *in vitro*^23^. In addition to platelet production, the expansion of HSCs is also a function of the TPO/Mpl system. Therefore, the question to be addressed is whether TPO function in pluripotent progenitor cells is conserved among vertebrates. The development of polyploidy in MKs is unclear^24^, although they can be artificially generated from embryonic stem cells, induced pluripotent stem cells, and fibroblasts^25^,^26^,^27^. The aim of this study was to characterize TPO/Mpl function in the development of mature/immature nucleated thrombocytes in non-mammalian vertebrates.

The structures and expression of mammalian TPO and Mpl are well understood. Mammalian TPO mRNA is expressed in hepatocytes, osteoblasts, kidneys, and the spleen^28^,^29^. Native human TPO circulates and maintains thrombopoiesis^30^, with constitutive hepatic expression of TPO and total Mpl mass, comprising MKs and platelets^31^. Posttranslational processing, including proteolytic cleavage, cleaves the C-terminal carbohydrate-rich domain of TPO, modulating its activity^32^. In addition, TPO mRNA expression is increased in the bone marrow and spleen of thrombocytopenic animals^33^. It is not clear if those particular mechanism are common to all vertebrates, and direct purification of TPO protein that provides physicochemical knowledge about native mammalian TPOs, has not been achieved in non-mammalian vertebrates^34^,^35^. Non-mammalian TPO genes have been identified and cloned from zebrafish and chicken^18^,^36^. Chicken TPO stimulates erythro- and thromboblastosis, and zebrafish TPO stimulates thrombopoiesis^18^,^36^. In the embryo of the African clawed frog, *Xenopus laevis*, rat TPO promotes expansion of haematopoietic progenitors, thus demonstrating cross-species TPO stimulation of haematopoieisis^37^.

*X. laevis* is one of the most popular animal models in embryology and physiology. We have directed our efforts to establishing a new animal model for the study of haematopoieisis^38^,^39^,^40^,^41^,^42^,^43^,^44^ and have investigated the physiological haematopoieisis response under a variety of environmental stress such as changes in temperature^41^,^45^. We recently reported that thrombocytic progenitors are localized in the liver and spleen of *X. laevis* and have a greater DNA content than do peripheral erythrocytes and thrombocytes^43^. Here, we describe the identification, cloning, and expression of biologically active *X. laevis* TPO (*xl*TPO) and Mpl (*xl*Mpl). *Xl*TPO induces the development of hepatic and splenic MKs as progenitors of nucleated thrombocytes in *X. laevis* and regulates the fate of peripheral Mpl-expressing thrombocytes via anti-apoptotic signalling. To our knowledge, this is the first report of the development of nucleated thrombocytes from MKs induced by the TPO/Mpl system.

## Results

### Identification and cloning of the *X. laevis* TPO and Mpl genes

**Figure 1:**
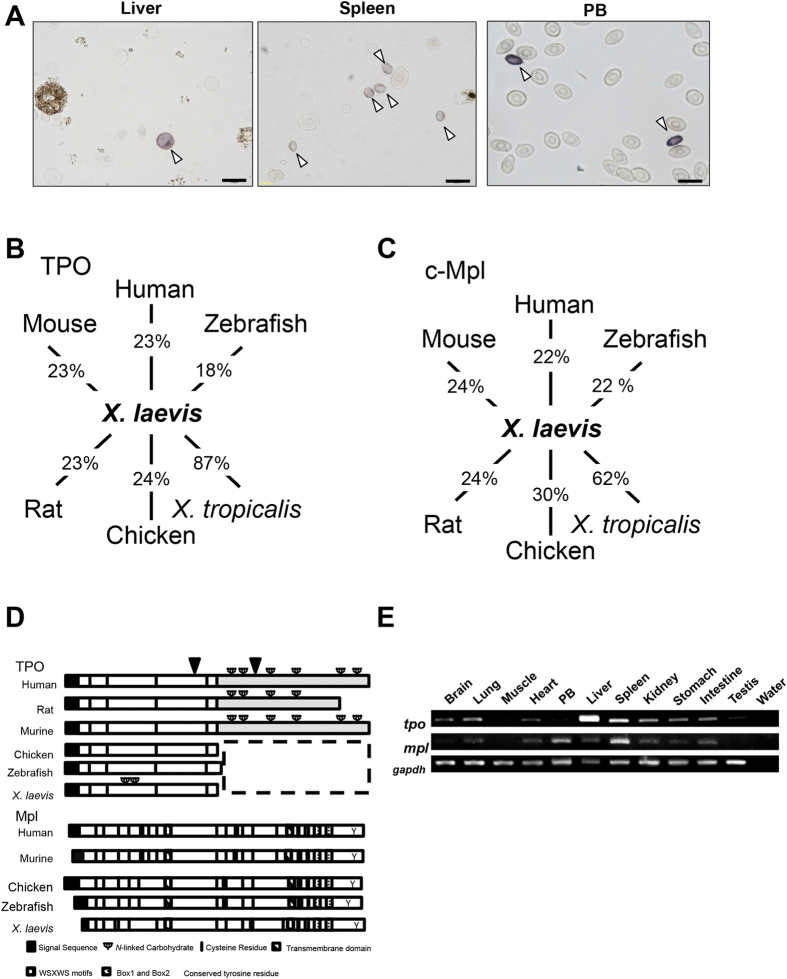
Conserved synteny homology between the *Xenopus tropicalis* and human TPO and Mpl loci and RNA expression of TPO and Mpl in *X. laevis* tissues. (**A**) Dissociated cells obtained from the *X. laevis* spleen, liver, and peripheral blood were immunostained for T12. Arrowheads indicate T12-positive cells. Scale bars represent 20 μm. (**B**) TPO similarity of the domain from the first to the fourth Cys residues in *X. laevis* and in human (23%), mouse (23%), rat (23%), chicken (24%), *X. tropicalis* (87%), and zebrafish (18%). (**C**) The extracellular region of *xl*Mpl shares homology with human (22%), mouse (24%), rat (24%), chicken (30%), zebrafish (22%), and *X. tropicalis* (62%). (**D**) Schematic diagram of human, rat, mouse, chicken, *X. laevis*, and zebrafish TPO and Mpl. Black boxes indicate signal sequences. Open box indicates the conserved erythropoietin (Epo)/Tpo domain. Black bars indicate the conserved cysteine residue. Grey box indicates the c-terminal TPO domain and putative cleavage sites are indicated by solid arrowheads. In Mpl, the vertical striped box shows haematopoietin domains with conserved WSXWS motifs; the dotted box shows Box1 and Box2, the shaded portion represents the transmembrane domain, and Y represents the conserved tyrosine residue. (**E**) RT-PCR analysis of *tpo* and *mpl* mRNA of *X. laevis* in selected organs. Uncropped gel images are shown in Supplementary Fig. S9.

We identified more than 60 putative orthologous of TPO by reference to public databases. Until now, there have been no comparative studies of molecular structure and function in other organisms (Supplementary Fig. S1). Among *X. laevis* hepatic and splenic T12^+^/CD41^+^ thrombocytic cells demonstrated in our previous study^43^, only hepatic large T12-positive cells are morphologically similar to MK in mammals (Fig. 1A). Therefore, we first hypothesized that thrombocyte progenitor in *X. laevis* originated from large cells, and attempted to clone TPO and Mpl in *X. laevis*. To identify the TPO sequence in *X. laevis*, we identified neighbouring genes in the human genome database. Polymerase RNA II DNA-directed polypeptide H (POLR2H) and chordin (CHORDIN) were then identified in the expressed sequence tag (EST) sequence of the *X. tropicalis* TPO locus (Supplementary Fig. S2A). cDNA encoding full-length *X. laevis* TPO was obtained by RT-PCR amplification from the adult *X. laevis* liver and spleen. The *X. laevis,* mammalian, zebrafish, and chicken TPOs were subsequently aligned (Fig. 1B). *X. laevis* shares an overall sequence identity of 87% with *X. tropicalis* TPO (Fig. 1B). The full-length *xl*TPO encodes a 153-amino acid-long protein; the non-mammalian TPO lacks the C-terminal domain. Mature *xl*TPO protein contains four cysteine residues (Cys-4, Cys-24, Cys-78, and Cys-97) that are conserved in human TPO (hTPO) at positions Cys-7, Cys-29, Cys-85, and Cys-151 (Supplementary Fig. S3). Zebrafish, chicken and *X. laevis* TPO lack a C-terminal half domain; however, the first to the fourth Cys residues of *xl*TPO are essential for biological activity in humans and rodents^30^. Therefore, we identified low but consistent sequence identities with the human (23%), mouse (23%), rat (23%), chicken (24%), and zebrafish (18%) homologs (Fig. 1B). The Mpl-neighbouring genes in the human genome database were identified as TIE-1 and CDC-20 (cell division cycle 20 homologue) (Supplementary Fig. S2B). The extracellular domain of Mpl contains highly conserved tyrosine and cysteine residues, WSXWS motifs, and Box1 and Box2 motifs necessary for intercellular signalling (Fig. 1D and Supplementary Fig. S4). The extracellular domain of *X. laevis* Mpl, which mediates ligand binding, shares homology with the human (22%), rat (24%), mouse (24%), chicken (30%), and zebrafish (22%) Mpl sequences (Fig. 1C). The low similarities of the *X. laevis* TPO and Mpl suggest substantial differences in the biological functions of the *X. laevis* TPO/Mpl system. mRNA expression of *xl*TPO and *xl*Mpl in the liver, spleen, kidney, and lung was confirmed by RT-PCR analysis; *xl*Mpl mRNA was detected in the spleen, kidney, intestine, and liver, and in thrombocytes in the peripheral blood (Fig. 1E).

### Biological activity of *xl*TPO

**Figure 2:**
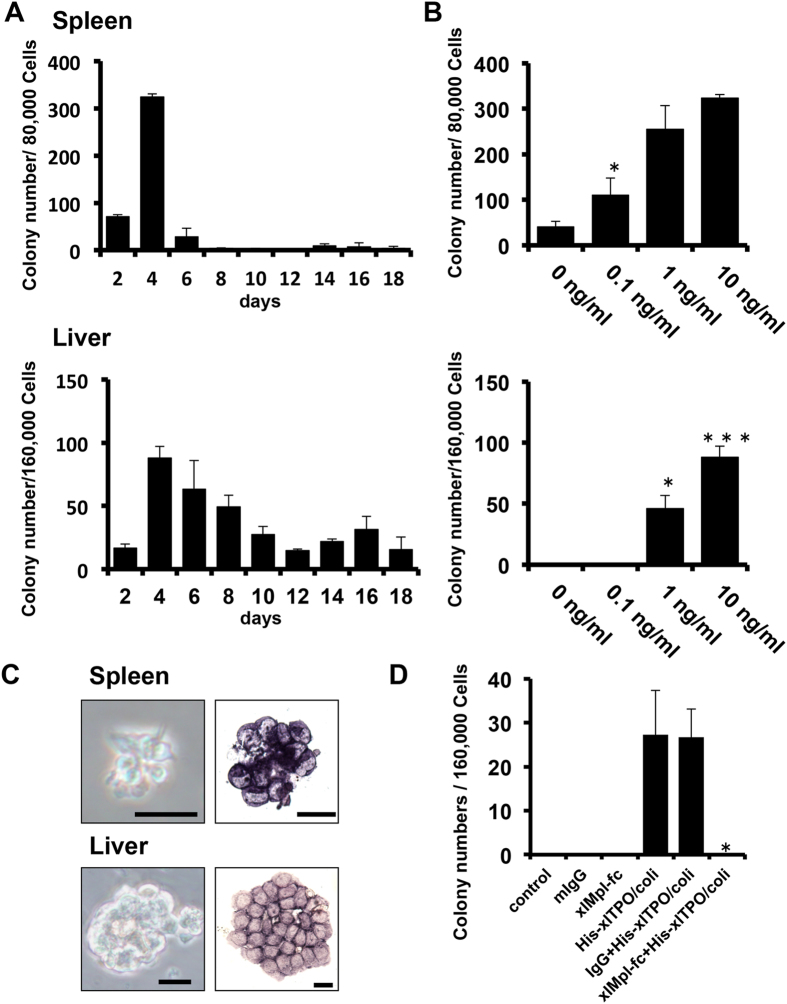
Biological activity of recombinant *X. laevis* TPO. (**A**) Colony-formation of spleen and liver cells in response to *xl*TPO. Upper panel shows the time course of colony formation by spleen cells; lower panel represents liver cells. (**B**) Dose-response effect of recombinant *xl*TPO on blast colony formation by splenic and hepatic cells. The upper panel shows spleen cell colony formation; the lower panel represents liver cells. Graphs represent means + SD, n = 3. *P < 0.05 vs. 0 ng/mL; **P < 0.05 vs. 0.1 ng/mL; ***P < 0.05 vs. 1.0 ng/mL. (**C**) Colony morphology. Left panels show colonies derived from *X. laevis* spleen and liver cells after 4 days culture in semisolid medium in the presence of *xl*TPO. Scale bars represent 20 μm. Right panels show the morphology of T12-stained colonies. (**D**) Hepatic cells were incubated with *xl*Mpl-Fc fusion protein or normal mouse IgG2a as a control in the presence of *xl*TPO, and cultured in semi-solid media. After 4 days, *xl*Mpl-Fc fusion protein inhibited colony formation; colonies formed in the presence of *xl*TPO alone or both *xl*TPO and IgG2a. Graphs represent means + SD, n = 3. *P < 0.05 vs. *xl*TPO or IgG2a + *xl*TPO stimulation.

Cells of the kidney, liver, and spleen were cultured in serum containing semisolid media with *xl*TPO. After 2 days, non-haemoglobinized colonies emerged from the hepatic and splenic cells and reached a peak on day 4; kidney cells did not yield colonies (Fig. 2A). *Xl*TPO (0.1–10 ng/mL) produced a concentration-dependent effect on thrombocytic cell proliferation in the spleen and liver (Fig. 2B). The colonies were T12-positive, indicating they were thrombocytic colonies, and they did not appear by erythropoietin (EPO) stimulation (Fig. 2C). To validate whether *xl*TPO stimulates proliferation with *xl*Mpl, we produced a fusion protein containing the extracellular domain of *xl*Mpl and the murine IgG2a Fc region. Thrombocytic colony formation was inhibited by *xl*Mpl-Fc in *xl*TPO-containing medium, whereas hepatic cells gave rise to thrombocytic colonies in the presence of *xl*TPO and IgG2a as the control (Fig. 2D). Thus, *xl*TPO stimulates thrombocytic cells via Mpl signalling. In addition, the effects of *xl*TPO activity in human Mpl were tested in cell proliferation assays using an hTPO-dependent cell line, UT-7/TPO. Despite low similarity in the primary structures of TPO and MPL (Fig. 1B,C), *xl*TPO stimulated proliferation of UT-7/TPO cells (Supplementary Fig. S5A,B), and JAK2 was tyrosine-phosphorylated (Supplementary Fig. S5C), similar to the effects of hTPO.

### Subpopulation of splenic and hepatic T12-positive cells by *xl*TPO stimulation

**Figure 3:**
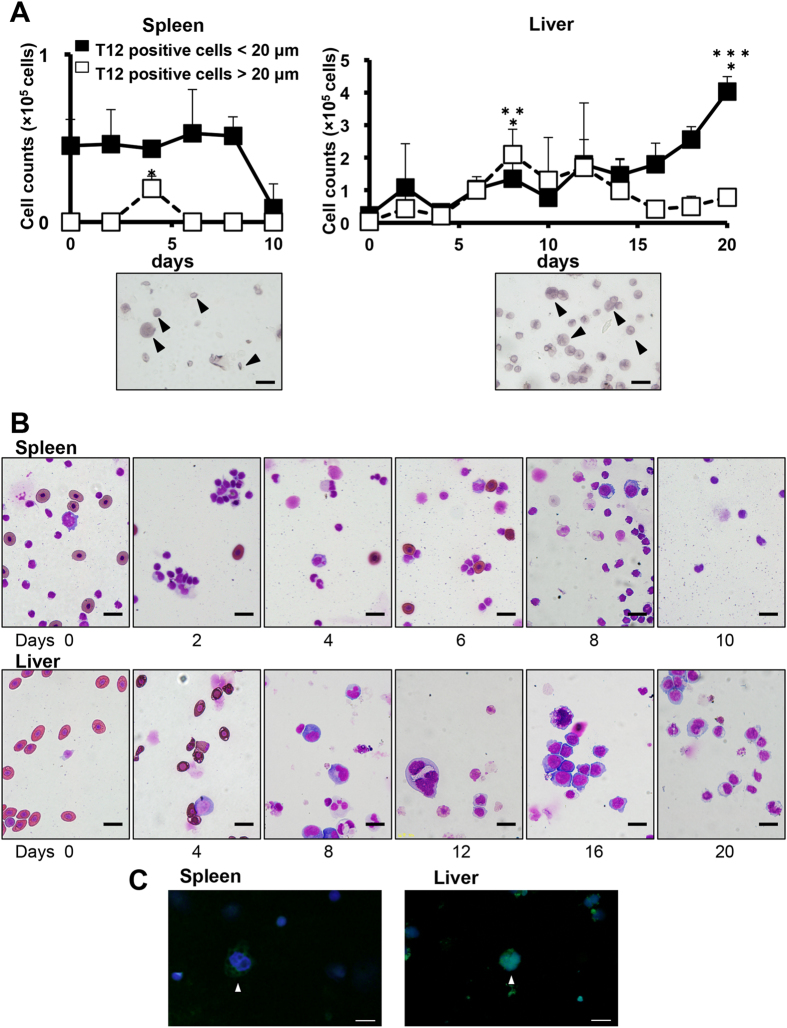
Proliferation and differentiation of hepatic and splenic thrombocytic cells. (**A**) Thrombocytic cell counts during liquid culture of spleen (Left) and liver (Right) cells in the presence of *xl*TPO. Cultured hepatic cells were cytocentrifuged onto slides, immunostained for T12, and counted. Open squares show T12-positive cells >20 μm; black squares indicate cells <20 μm. *P < 0.05 vs. day 0; **P < 0.05 vs. day 0; ***P < 0.05 vs. day 20 in T12-positive large cells. The lower panel shows large and small T12-positive cells derived from splenic and hepatic cells after 4 and 8 days of culture in medium containing *xl*TPO. Scale bars represent 20 μm. Graphs represent means + SD, n = 5. (**B**) After 2 to 20 days culture in the presence of *xl*TPO, hepatic or splenic cells were cytocentrifuged onto glass slides and stained with May-Grunwald-Giemsa (MGG). Scale bar indicates 20 μm. (**C**) The morphology of T12-positive splenic or hepatic cells after culture in the presence of *xl*TPO for 4 days. Immunostaining for T12 was performed. Biotinylated T12 was detected by streptavidin-conjugated Alexa Fluor 488 (green). Nuclei were counterstained with Hoechst 33342. Bars represent 20 μm.

In *X. laevis*, PHZ induced acute haemolysis, erythrocyte counts reached a nadir within 8 days, and the number of immature haematopoietic progenitors increased in the liver and spleen^39^. Anaemic serum samples were obtained 4 days after PHZ administration to obtain the maximal colony-forming activity. Hepatic or splenic cells from PHZ-induced anaemic *X. laevis* were cultured in a semisolid culture system in the presence of serum collected at day 4. Thereafter, T12 and *o*-dianisidine co-positive colonies emerged (Supplementary Fig. S6A), suggesting the presence of bipotent thrombocyte/erythrocyte progenitors in the liver and spleen of *X. laevis*, as in chicken^46^. We also observed erythrocyte colonies, thrombocytic colonies, and colonies of non-haemoglobinized cells (Supplementary Fig. S6 B–D). To characterize the thrombocyte progenitors, hepatic and splenic cells were stimulated by *xl*TPO (5 ng/mL) in foetal calf serum (FCS) containing 0.8 × diluted alpha minimum essential medium (dα-MEM). Although the number of splenic cells decreased, hepatic cells increased in the presence of *xl*TPO (Supplementary Fig. S7A). T12-positive cells were counted every second day. After 4 days, the T12-positive cells appeared as large, multinucleated spheres (approximately 25 μm in diameter); the peripheral spindle-shaped elliptic thrombocytes in *X. laevis* were approximately 20 μm × 6 μm in cytocentrifuge preparations (Fig. 3A,B). These T12-positive cells were grouped according to size: large (20–30 and 30–50 μm in diameter) and small (<20 μm in diameter). The *xl*TPO-induced T12-positive round cells in spleen were only observed 4 days after stimulation and had died by day 10. While in the liver, T12-positive large cell counts increased in response to *xl*TPO and peaked at day 8; thereafter, the counts began to decrease. Small T12-positive cells increased about 40-fold from day 0 to day 20 (Fig. 3A). The T12-positive large cells had multi-lobular nuclei and features similar to those of mammalian MKs (Fig. 3C). The nucleus-cytoplasm ratio (N/C ratio) in the subpopulation of cells having a diameter of 10–20 μm (10–20 μm fraction) was higher than those 20–30 and 30–50 μm fractions, indicating the T12 recognizes the different developmental stages of thrombocytes. Additionally, the N/C ratio of 10–20 μm T12-positive cells in the spleen was higher than those in the 20–30 and 30–50 μm fractions. On the other hand, the ratio of the 20–30 μm T12-positive cell fraction in the liver was higher than those in the 10–20 and 30–50 μm fractions, suggesting that T12-positive cells at different developmental stages resided in the liver and the spleen (Supplementary Fig. S7B,C).

### Characterization of MKs in *X. laevis*

**Figure 4:**
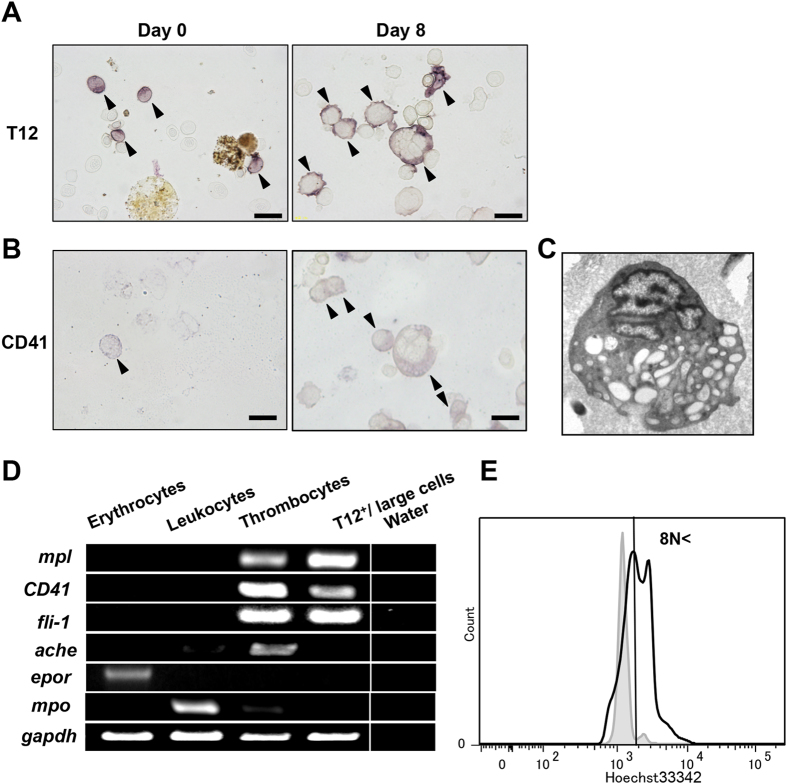
Characterization of large MKs. (**A**) The morphology of pre-culture hepatic cells as demonstrated with T12 immunostain. After eight days in the presence of *xl*TPO, the hepatic cells were again immunostained for T12. Arrowheads indicate T12^+^ cells. Bars represent 20 μm. (**B**) The 8-day cultured cells were also immunostained with CD41 polyclonal antibody. Arrowheads indicate CD41^+^ cells. Bars represent 20 μm. (**C**) Transmission electron micrographs of MK on day 8. (**D**) Expression profiles of *X. laevis* Mpl, CD41, Fli-1, AchE, EPOR, MPO, and GAPDH mRNA in peripheral blood cells and MK. Peripheral erythrocytes, leukocytes, and thrombocytes were collected and prepared as described in Materials and Methods. (**E**) Ploidy of MKs after *xl*TPO stimulation for eight days versus normal peripheral blood as a control.

MKs in *X. laevis* expressed T12 and CD41 and appeared when hepatic or splenic cells were stimulated by recombinant *xl*TPO (Fig. 4A,B). After 8 days of culture, T12-positive large cells were collected by cytometry, and the cytosolic structures were observed by transmission electron microscopy. The vacuolar system in T12-positive large cells closely resembled the surface-connecting system of MKs and platelets in mammals, and the granules in T12-positive large cells in *X. laevis* also resemble the dense granules in MKs and platelets in mammals (Fig. 4C). RT-PCR showed the T12-positive large cells express Mpl, CD41, and Fli-1 but not acetylcholine esterase (AChE), EPOR, or myeloperoxidase (MPO) (Fig. 4D). The T12-positive large cells at 8 days of culture were stained with Hoechst 33342 for flow cytometry, which showed the fluorescence intensities of the T12-positive large cells were 2- to 4-fold higher than those of mature thrombocytes (Fig. 4E). On the basis of these findings, we defined T12-positive large cells as MKs in *X*. *laevis*, and the data demonstrated that MKs are involved in thrombocytic lineage.

### Differentiation of mature thrombocytes

**Figure 5:**
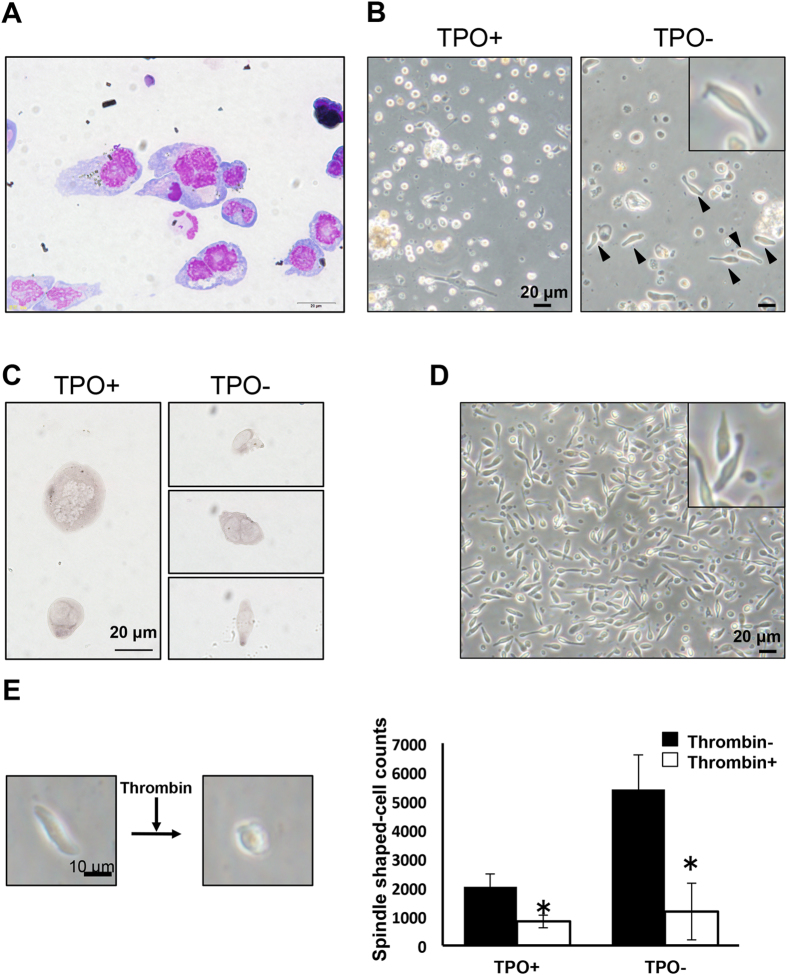
Maturation of thrombocyte-like cells from MKs. (**A**) MKs were enriched by density-gradient centrifugation. The 50% layer was collected and stained with MGG. (**B**) Enriched MKs were cultured in the presence or absence of *xl*TPO. After two days suspension culture in the absence of *xl*TPO, spindle-shaped thrombocyte-like cells were observed (solid arrowheads and inset). Bars represent 20 μm. (**C**) The morphology of cultured hepatic cells. Enriched MKs were cultured for two days in the presence or absence of *xl*TPO; hepatic cells were cytocentrifuged onto slide glass and stained with T12. (**D**) The morphology of peripheral thrombocytes in suspension after two days (inset). (**E**) Whole cultured thrombocytes were incubated with or without thrombin, and the proportion of spindle-shaped thrombocytes was calculated. Left panels show the changing morphology of cultured thrombocyte-like cells. Graphs represent means + SD, n = 6. *P < 0.05 vs. thrombin^-^.

Cultured MKs were enriched by Percoll density-gradient centrifugation (Fig. 5A) and cultured in the presence or absence of *xl*TPO. After 2 days, spindle-shaped cells appeared in MKs cultured without *xl*TPO (Fig. 5B). These cells were T12-positive, suggesting they have features of peripheral thrombocytes (Fig. 5C). The peripheral thrombocyte fraction was collected by density-gradient centrifugation and cultured in dα-MEM containing 20% FCS. After 4 days, the thrombocytes had acquired a barbell morphology similar to cultured thrombocytic cells (Fig. 5D and Supplementary Fig. S8A). To determine whether these cells were functional thrombocytes, a thrombocyte activation assay was performed. After stimulation with thrombin, spindle-shaped thrombocytic cells were counted; upon thrombin activation, their morphology changed (Fig. 5E). These results were consistent with the functions of peripheral thrombocytes.

### TPO-Mpl signalling in peripheral thrombocytes

**Figure 6:**
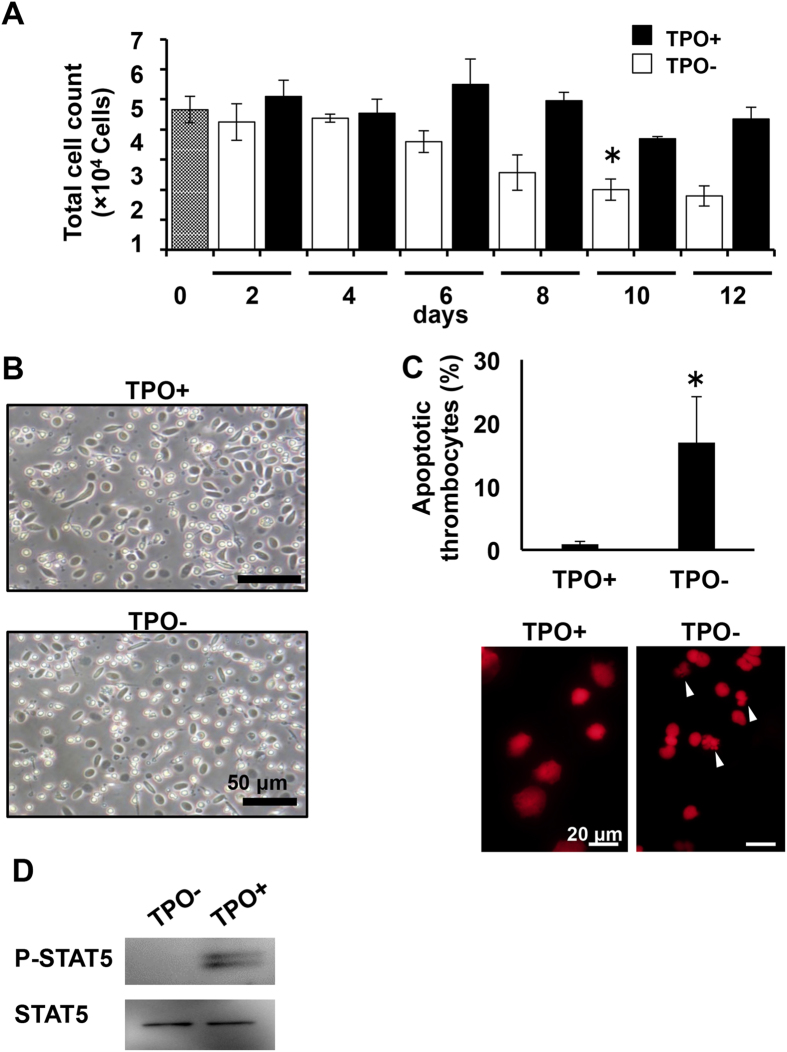
Functions of *xl*TPO–*xl*Mpl signalling in peripheral thrombocytes. (**A**) Peripheral thrombocytes were collected by density-gradient centrifugation and cultured in dα-MEM with *xl*TPO. Thrombocyte viability was assessed by trypan blue staining. Thrombocyte numbers are indicated after stimulation with 10 ng/mL *xl*TPO (black bars) and without stimulation (white bars). Graphs represent means ± SD, n = 3. *P < 0.05 vs. day 0. (**B**) Thrombocyte morphology in suspension in the presence (upper panel) or absence (lower panel) of *xl*TPO for 8 days of culture. (**C**) Representative image of apoptotic cells detected by Propidium iodide nuclear staining. Apoptotic cells were counted after 8 days of culture. Apoptotic nuclei are marked with arrows (Bar, 20 μm). Graphs represent means + SD, n = 3. *P < 0.05 vs. TPO+. (**D**) STAT5 phosphorylation in thrombocytes. Western blots of P-STAT5 and STAT5 in the presence or absence of *xl*TPO. Uncropped gel images are shown in Supplementary Fig. S9.

**Figure 7:**
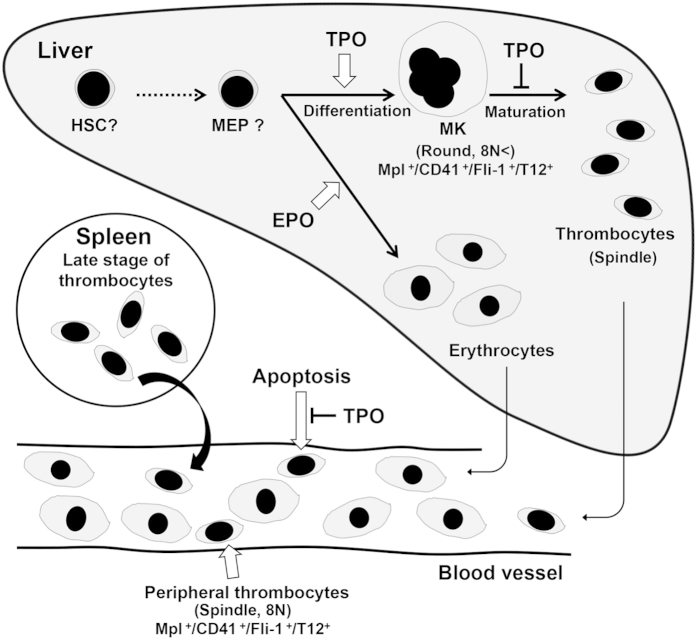
Schematic model of thrombopoiesis in *X. laevis*. Thrombocyte progenitors mainly resided in the liver, where they localized in the sinusoid and differentiated to MKs with *xl*TPO stimulation. Final thrombocyte production from MKs inhibited by *xl*TPO. Peripheral thrombocytes expressed Mpl and *xl*TPO regulated thrombocyte viability.

The Mpl gene was also expressed in peripheral thrombocytes. To examine TPO/Mpl signalling in *X. laevis*, peripheral thrombocytes were collected and cultured in the presence of *xl*TPO. *Xl*TPO enhanced thrombocyte viability by 50% relative to untreated thrombocytes at day 10 (Fig. 6A). The irregular shape of the non-stimulated thrombocytes indicated the cells were dead (Fig. 6B). We then tested apoptosis induction by analysing karyorrhexis. Unlike thrombocytes treated with *xl*TPO, untreated thrombocytes exhibited increased rates of apoptosis (Fig. 6C). *Xl*TPO (0.1–10 ng/mL) also produced a concentration-dependent effect on antiapoptosis of peripheral thrombocytes after 8 days of culture (Supplementary Fig. S8B). In addition, *xl*TPO activated STAT5 phosphorylation (Fig. 6D), indicating the role of *xl*TPO as an anti-apoptotic regulator in peripheral thrombocytes. The proposed mechanism of *xl*TPO function in the liver, spleen, and peripheral blood is summarized in Fig. 7.

## Discussion

Mammalian platelets are derived from the cytoplasm of MKs through endocytosis, primarily induced by TPO, which mediates MK maturation and stem cell expansion. In this study, we explored the origin of nucleated thrombocytes in the circulation and the functional role of the *X. laevis* TPO/Mpl system in the development of nucleated thrombocytes. The N-terminal region of hTPO is homologous to human EPO, with which it shares 23% sequence identity. The *xl*TPO sequence also overlaps with *X. laevis* EPO (*xl*EPO) by 30%, although *xl*TPO stimulates the production of thrombocytes, *i.e.*, thrombopoiesis, and *xl*EPO stimulates erythropoiesis^39^. Therefore, the function of EPO is distinct from that of TPO, at least in amphibians. *Xl*TPO stimulates phosphorylation of cellular proteins in human UT-7/TPO cells (Supplementary Fig. S5C), demonstrating a certain tertiary structure in the TPO-Mpl binding is partly shared, despite differences in the primary structure (Fig. 1B,C and Supplementary Fig. S1). The conservation of Cys residues also suggests receptor recognition is conserved. This finding is crucial to understanding the evolution of TPO signalling.

The C-terminal region of hTPO contains multiple *N*-glycosylation sites that mediate secretion of the protein^47^, and circulating native hTPO comprises this full-length form^48^. In contrast, *xl*TPO lacks the C-terminal domain of mammalian TPOs but does possess potential *N*-glycosylation sites in the N-terminus. In *X. laevis*, *xl*TPO is produced in the liver where thrombocytes develop, in an analogous way to *X. laevis* erythropoiesis^39^,^40^. In contrast, *xl*TPO mRNA is highly expressed in the lung where there are no Mpl-expressing cells reside. Therefore, *xl*TPO likely circulates in the blood and is stabilized by the sugar chain, which influences the lifespan of circulating thrombocytes (Fig. 6). Human TPO primes platelet aggregation induced by shear stress and various agonists^49^, and blood levels are regulated by the platelet mass through TPO binding to Mpl on the platelet surface under constitutive hepatic expression of TPO mRNA^31^. In this study, *xl*TPO stimulated phosphorylation of STAT5 and anti-apoptosis in peripheral thrombocytes *in vitro* (Fig. 6C,D). Phosphorylation of STAT5 induced the expression of the anti-apoptotic factors *bcl-xL* and *pim-1*^50^. Therefore, circulating *xl*TPO might suppress apoptosis in peripheral thrombocytes through bcl-xL signalling and might regulate the fate of peripheral thrombocytes.

Colony formation in the presence of *xl*TPO and *xl*EPO revealed thrombocyte and erythrocyte progenitors in the liver and spleen of *X. laevis* (Fig. 2)^40^. We have previously reported that peripheral thrombocytes and T12-positive cells in the liver have 2- and 4-fold greater DNA content than do erythrocytes, whereas polyploidization is the same in splenic T12-positive cells and peripheral thrombocytes^43^. While hepatic cells could be cultured for more than 2 weeks in the presence of *xl*TPO stimulation, splenic cells could not (Supplementary Fig. S7A). In addition, cellular distribution of 10–20 μm-T12 positive cell in the spleen was higher than the liver (Supplementary Fig. S7B), suggesting that thrombocyte progenitors are present at different developmental stages in the liver and spleen. Functional thrombocytes are produced from CD41- and T12-positive MKs, primarily in the liver, whereas morphologically mature thrombocytes localize in the spleen^43^. Although thrombocyte progenitors reside in the spleen and liver, thrombocyte progenitors are more immature in the liver than in the spleen. Thus, the *X. laevis* liver niche regulates MKs maturation, whereas the spleen niche is superior at a later stage, when it comprises peripheral thrombocytes. In adult mammals, haematopoietic progenitors reside in the microenvironment of the bone marrow composed of osteoblast, endothelial, and stromal cells that regulate stem cell quiescence and differentiation^51^. The spleen and liver niches in *X. laevis* comprise soft tissue lacking in osteogenesis, and both organs are potential destruction sites of thrombocytes; the difference may reveal underlying factors of terminal differentiation.

Only non-mammalian HSCs have been identified in ginbuna crucian carp and zebrafish^52^,^53^. In mice, TPO regulates HSC quiescence and interaction with the osteoblastic niche^54^, and HSCs are absent in Mpl-deficient mice^55^. A murine Mpl derivative lacking the distal 60 amino acids revealed the essential nature of the membrane-proximal region for maintenance of HSC activity^56^; *xl*Mpl also possesses this membrane-proximal region (Fig. 1D). hTPO stimulates *in vitro* proliferation of progenitor cells for more than 2 weeks; *xl*TPO also stimulates proliferation of *X. laevis* hepatic cells for more than 2 weeks, suggesting that the presence of immature and earlier haematopoietic progenitors in *X. laevis* needs to be addressed in future studies. Recent studies have found that heterogeneous HSCs can directly differentiate into megakaryocyte progenitors^57^. Although we could not evaluate the HSCs in *X. laevis*, it is important to identify the main pathway to produce the thrombocytes for development.

Our study demonstrated that nucleated thrombocyte progenitors in *X. laevis* resemble platelet progenitors in mammals. Both have multilobed nuclei and produce haemostatic cells activated by thrombin, suggesting the platelets evolved from nucleated spindle-shaped elliptic thrombocytes. In *X. laevis*, blood capillaries do not develop as in mammals^58^ and a proplatelet formation system is unnecessary. The number of peripheral thrombocytes in *X. laevis* (30 g) is approximately 3 × 10^7^ cells; moreover, the ratio of peripheral thrombocytes to total thrombocytes is lower in *X. laevis* than in mammalian species^43^. The DNA content of mammalian MKs increases from 2 N to 256 N, and 4000–8000 platelets may be produced from a single MK^59^. In this study, the DNA content of *X. laevis* MKs increased from 4N (allotetraploid) to 32N, and a single MK divided at least twice and produced 1–4 thrombocytes.

The *X. laevis* model will provide new insights in the field of comparative haematology with the added support of the *X. laevis* genome database [Horb M *et al.* *Xenbase*. 2014 Xenopus Community White Paper. Improvement of Xenopus antibody resources. 2014. Available at: http://www.xenbase.org/community/xenopuswhitepaper.do. Accessed June 10, 2015]. Our data showed the function of *xl*TPO in thrombocyte production in the liver and spleen, and as an inducer of anti-apoptosis in peripheral thrombocytes. We demonstrated the derivation of thrombocytes from MKs induced by TPO/Mpl signalling in *X. laevis* and conclude that the process of platelet production is partially conserved in the mechanism of non-mammalian thrombocyte production.

## Materials and Methods

All animal experiments were performed in accordance with the approved protocols and guidelines of the Steering Committee for Animal Experimentation at Waseda University.

### Animals

Wild-type male *X. laevis* (10–30 g) were purchased from Aquatic Animal Supply (Misato, Saitama, Japan) and housed in a 20-L aquarium on a 12-h light/dark cycle at 22 °C.

### Isolation of the *X. laevis* TPO and Mpl genes

To identify the *xl*TPO and *xl*Mpl genes, we searched neighbouring genes at the TPO locus (NCBI gene ID 4352) and Mpl locus (NCBI gene ID 7066) in the human genome database. POLR2H and CHORDIN were identified at the TPO locus, and tyrosine kinase with immunoglobulin and epidermal growth factor homology domains TIE-1 and CDC-20 were identified at the Mpl locus. These were then searched against the NCBI Homologene or BLAST database for *X. tropicalis*. For *xl*TPO, we found an EST sequence in the *X. tropicalis* TPO locus. The EST sequence was obtained from GenBank (accession number: DR881950). For *xl*Mpl, we searched for conserved Mpl sequences by TBLASTN of the *X. tropicalis* Mpl locus with the TIE-1 and CDC-20 genes. The program GENESCAN (http://genes.mit.edu/GENSCAN.html) was used to predict the exons and coding sequences.

### *X. laevis* RNA preparation and DNA sequencing

Total RNA was extracted from various adult *X. laevis* tissues using TRIZOL reagent (Invitrogen). The purified total RNA was reverse-transcribed into cDNA using ReverTraAce (Toyobo). To generate the putative *xl*TPO cDNA fragment, RT-PCR of total RNA from spleen and liver cells was performed using primers specific for *X. tropicalis* TPO (*xt*TPO)_Fw2 (5′-TCGATTCGCTCATATTCTGC-3′) and *xt*TPO_Re2 (5′-TGGCAAGGTACAGTGTAGTCCA-3′) and Ex *Taq* polymerase or *pfu* polymerase (Takara). Primers were complementary to the 5′ and 3′ edges of the EST sequence obtained from the *X. tropicalis* genome database. The same primer pair was used to analyse TPO expression in various *X. laevis* tissues. The obtained cDNA fragment was cloned into pGEM-T Easy (Promega) and sequenced on an Applied Biosystems 3130 Genetic Analyzer with dye-terminator chemistry.

### Sequence analysis, alignment

Protein sequences for the mammalian TPO and Mpl were retrieved from the NCBI database. The signal peptide of *xl*TPO was predicted by SignalP and compared to the reported amino acid sequences of other mammalian species. Alignments of the *X. laevis* and mammalian TPO and Mpl sequences were generated with ClustalX software.

### Morphology and staining

Cells were collected and stained with MGG and immunostained with T12 and CD41 as previously described^38^,^43^.

### Western blotting

Thrombocytes were collected by gradient density centrifugation, washed twice with 0.8 × Dulbecco’s modified phosphate-buffered saline with EDTA to remove Mg^2+^ and Ca^2+^ ions. Total thrombocyte protein was extracted in M-PER lysis buffer (ThermoFisher) containing protease and phosphatase inhibitors (Roche Applied Science). SDS polyacrylamide gel-electrophoresis (PAGE) was performed as previously described^43^. Lysates were prepared and 3 μg of total lysate were quantified and separated by SDS-PAGE; gels were blotted onto polyvinylidene difluoride membranes (Millipore) and stained polyclonal antibodies to STAT-5 (1:200 dilution, Santa Cruz) and phosphorylated STAT-5 (1:1000 dilution, BD Biosciences). Antibody binding was detected by incubation with a horseradish peroxidase-labelled secondary antibody, followed by chemiluminescence detection (ECL-Plus; Amersham Pharmacia Biotech).

### RT-PCR

Tpo, Mpl, CD41, Fli-1, Erythropoietin receptor (EPOR), and *X. laevis* glyceraldehyde-3-phosphate dehydrogenase (gapdh; control) expression levels were measured by RT-PCR for 35 cycles (LabRepCo) with the following primer sequences for tpo and mpl (primer sequences for CD41, Fli-1, and EPOR gene are described previously)^43^: *tpo*-Fw, 5′-AGAGAAAATCGGCACAATGC-3′ and *tpo*-Re, 5′-GGGCTTTCTCTCAGACGATG-3′, and *mpl*-Fw, 5′-CCTTTGGATGGGTTTTGGG-3′ and *mpl*-Re, 5′-TTATCCAGCCAGCACTTGCA-3′.

### *In vitro* colony assay and liquid cell culture

Haematopoietic cells were isolated from the liver and spleen of *X. laevis*. These cells were filtered (BD Biosciences Clontech) and washed four times with dα-MEM (Invitrogen). The cells were cultured at 0.8 × 10^5^ cells/mL in 35-mm plastic Petri dishes (Corning) in 2 mL dα-MEM containing 20% heat-inactivated FCS, 0.8% methylcellulose (Shin-Etsu Chemical), and 100 μg/mL streptomycin (Invitrogen) and 100 μg/mL kanamycin (Invitrogen). Escherichia coli BL21(DE3) was transformed with plasmid pET19b (Takara), which encodes a full-length cDNA copy of *xl*TPO encoding the enterokinase inserted between NdeI and BamHI sites, and *xl*TPO was expressed by this plasmid. Stimulators were added where appropriate, and the cells were incubated in 5% CO_2_ at 23 °C. Colony formation was assessed on alternate days from day 2 to day 18. Colonies were picked with a 20-μL pipet tip, suspended in dα-MEM containing 10% FCS, and centrifuged (400 rpm). The samples were stained with various dyes in triplicate experiments. Error bars in figures depict standard errors. Differences were considered significant at the 95% confidence level (P < 0.05). We produced a soluble *xl*Mpl-Fc fusion protein (*xl*Mpl-Fc) containing the extracellular domain of *xl*Mpl (aa 11–246) and the murine IgG2a Fc region in HEK293 cells. To verify *xl*TPO/*xl*Mpl signalling, 1 ng/mL *xl*TPO was incubated with 500 ng/mL *xl*Mpl-Fc for 30 min and added to hepatic cells in semisolid culture to assess neutralization; mouse IgG2a (Dako) was incubated with *xl*TPO as the control. Hepatic or splenic cells were cultured as the liquid suspension in dα-MEM containing 20% FCS and *xl*TPO (5 ng/mL). Cells were counted and suspended at 5.0 × 10^5^ cells/mL, then cultured with 500 μL dα-MEM containing 10% FCS supplemented with 100 μg/mL streptomycin and 100 μg/mL kanamycin for 20 days at 23 °C.

### Statistical analysis

Results are presented as means ± SE. Statistical analysis included analysis of variance and Student’s *t*-test. A P-value of 0.05 or less was considered statistically significant.

## Additional Information

**How to cite this article**: Tanizaki, Y. *et al.* Thrombopoietin induces production of nucleated thrombocytes from liver cells in *Xenopus laevis*. *Sci. Rep.* **5**, 18519; doi: 10.1038/srep18519 (2015).

## Supplementary Material

Supplementary Information
