# Supplementary material for: Thrombopoietin induces production of nucleated thrombocytes from liver cells in Xenopus laevis
Source: Sci Rep. 2015 Dec 21;5:18519. doi: 10.1038/srep18519 (PMC4685256; doi:10.1038/srep18519)
Supplement: Supplementary Information [file srep18519-s1.pdf]

Supplemental Information

Thrombopoietin induces production of nucleated thrombocytes from liver cells in *Xenopus laevis*

Yuta Tanizaki[1], Megumi Ichisugi[2], Miyako Shimoji-Obuchi[3], Takako Ishida-Iwata[3], Ayaka Mogi-Tahara[2], Mizue Ishikawa-Meguro[2], and Takashi Kato[1,2,3]

[1]Department of Biology, School of Education, Waseda University, 2-2 Wakamatsu, Shinjuku, Tokyo, 162-8480, Japan, [2]Integrative Bioscience and Biomedical Engineering, Graduate School of Advanced Science and Engineering, Waseda University, 2-2 Wakamatsu, Shinjuku, Tokyo 162-8480, Japan; [3]Education and Integrated Arts and Sciences, Waseda University; 2-2 Wakamatsu, Shinjuku, Tokyo 162-8480, Japan

*Author for correspondence: Takashi Kato

Integrative Bioscience and Biomedical Engineering, Graduate School of Advanced Science and Engineering, Waseda University

2-2 Wakamatsu, Shinjuku, Tokyo 162-8480, Japan

Phone: +81 (3) 5369-7309, Fax: +81 (3) 3355-0316, Email: tkato@waseda.jp

Supplementary Figure 1

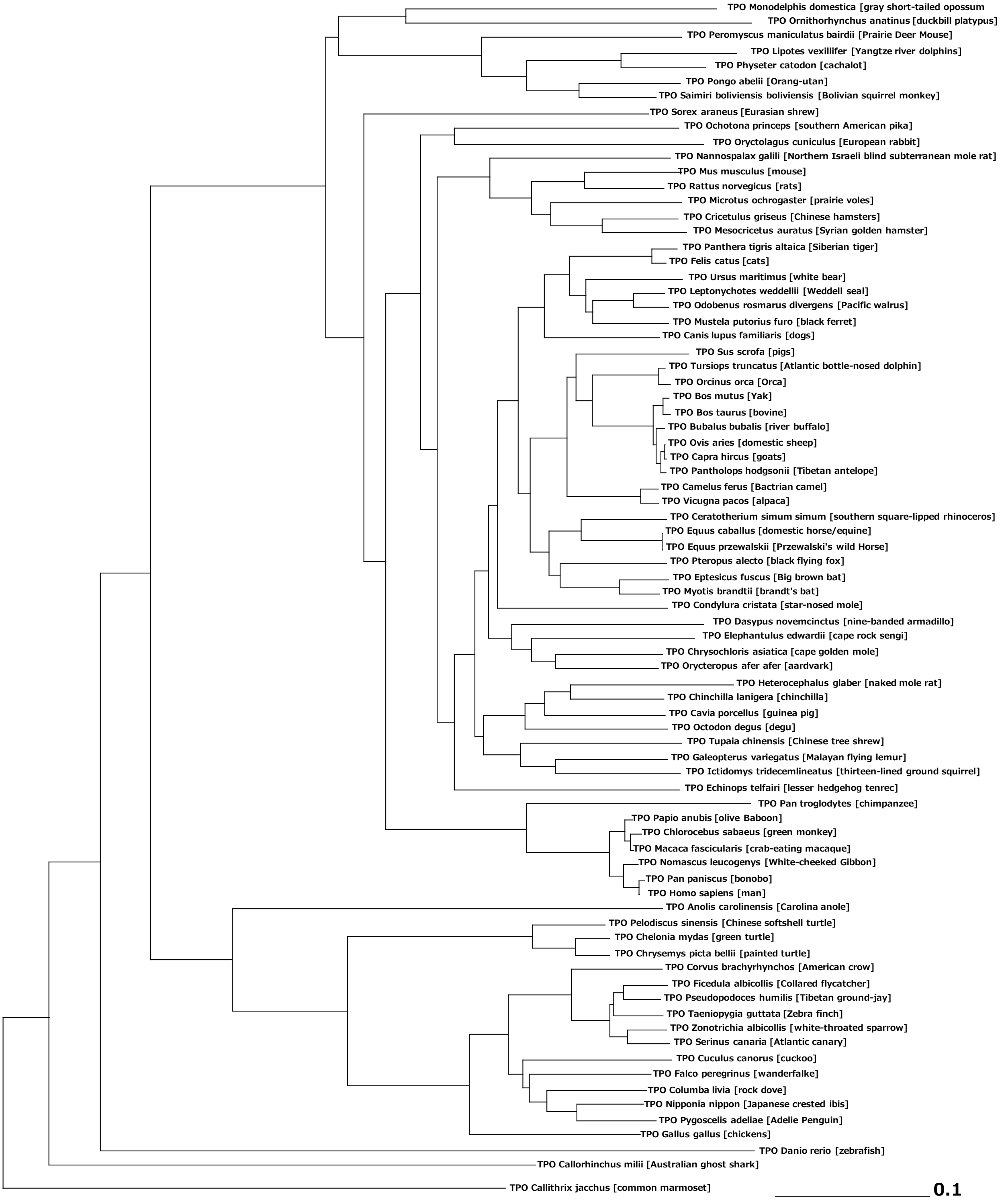

Supplementary Figure 1. Phylogeny of putative thrombopoietin. Phylogeny analyses were performed by use of Clustal Omega and Clustal Phylogeny for proteins, provide through the analysis tool web services from the EMBL-EBI (http://www.ebi.ac.uk/Tools/msa/clustalo/). The deduced sequences of TPO were aligned with the program Clustal Omega and a tree was calculated using TreeViewX software[1-3].

1. Sievers, F. *et al.* Fast, scalable generation of high-quality protein multiple sequence alignments using Clustal Omega. *Molecular Systems Biology* **7,** (2011).
2. Goujon, M. *et al.* A new bioinformatics analysis tools framework at EMBL-EBI. *Nucleic Acids Res.* **38,** (2010).
3. McWilliam, H. *et al.* Analysis Tool Web Services from the EMBL-EBI. *Nucleic Acids Res.* **41,** (2013).

Supplementary Figure 2

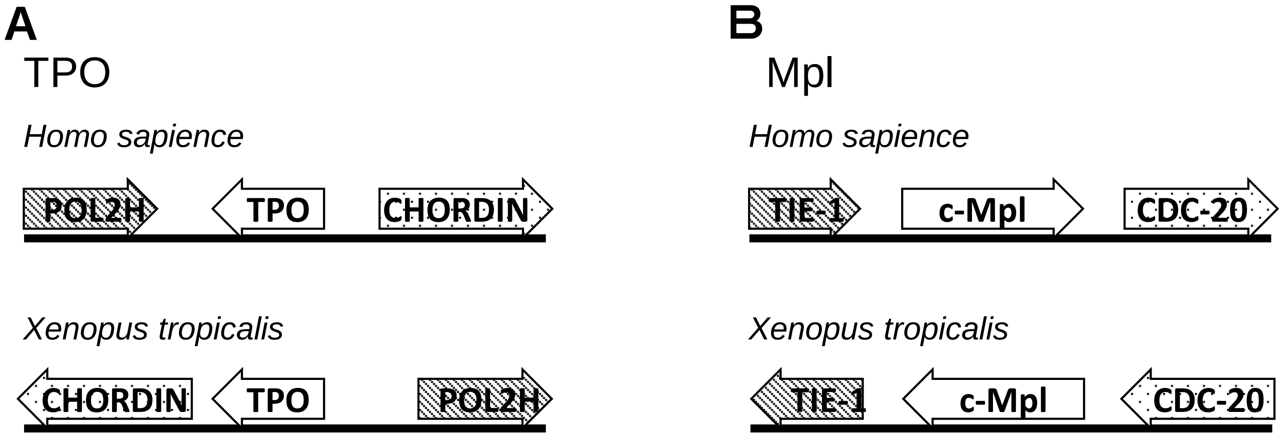

Supplementary Figure 2. Conserved synteny and homology between the *X. tropicalis* and human TPO and Mpl loci. (A) RNA polymerase II H (POL2H) and Chordin, mapped in the hTPO locus, were identified by NCBI Homologene or BLAST search in the *X. tropicalis* genome database. The synteny of POL2H, CHORDIN, and TPO at the human, mouse, chicken, and *X. tropicalis* TPO loci was conserved, although their order and direction of transcription has been shuffled (B) TIE-1 and CDC-20 in *xl*Mpl were identified by NCBI Homologene or BLAST in the *X. tropicalis* genome database. The synteny of TIE-1, CDC-20, and Mpl at the human, mouse, chicken, and *X. tropicalis* Mpl loci was also conserved, although their order and direction of transcription has been shuffled.

Supplementary Figure 3

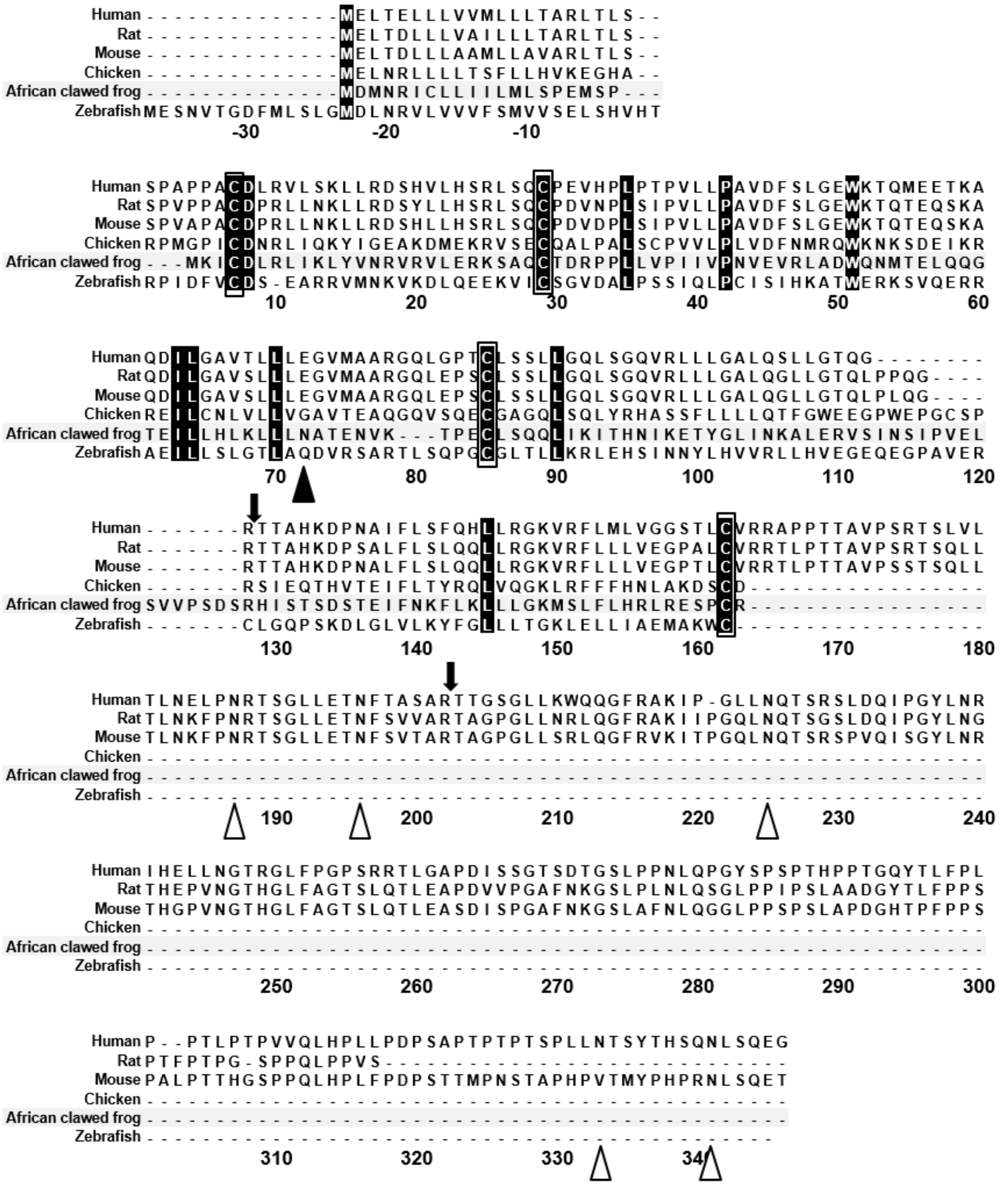

Supplementary Figure 3. Homology of TPOs from various vertebrate species. Amino acid sequence homology of mature TPOs from human, rat, mouse, chicken, *X. laevis*, and zebrafish. Numbers represent positions from the N-terminal end of mature *xl*TPO. The predicted signal peptide is shown at the top and indicated in lowercase letters. Identical amino acid residues are shaded black. Cysteine residues that form possible disulfide bridges in *xl*TPO are boxed. Positions of potential *N*-linked carbohydrate on *xl*TPO and hTPO are indicated by solid and open arrowheads, respectively. Potential sites for *N*-glycosylation are conserved in human, rat, and murine TPOs, whereas zebrafish, chicken, and *X. laevis* TPOs lack a C-terminal domain. The thrombin cleavage site in hTPO is indicated by an arrow. The tertiary structure of human TPO has been solved as an antiparallel four-helix bundle fold. TPO sequences were obtained from GenBank (accession numbers: human, AAI30323; rat, BAA06906; mouse, AAA40436; chicken, AAT45554; zebrafish, NP_001137225).

Supplementary Figure 4

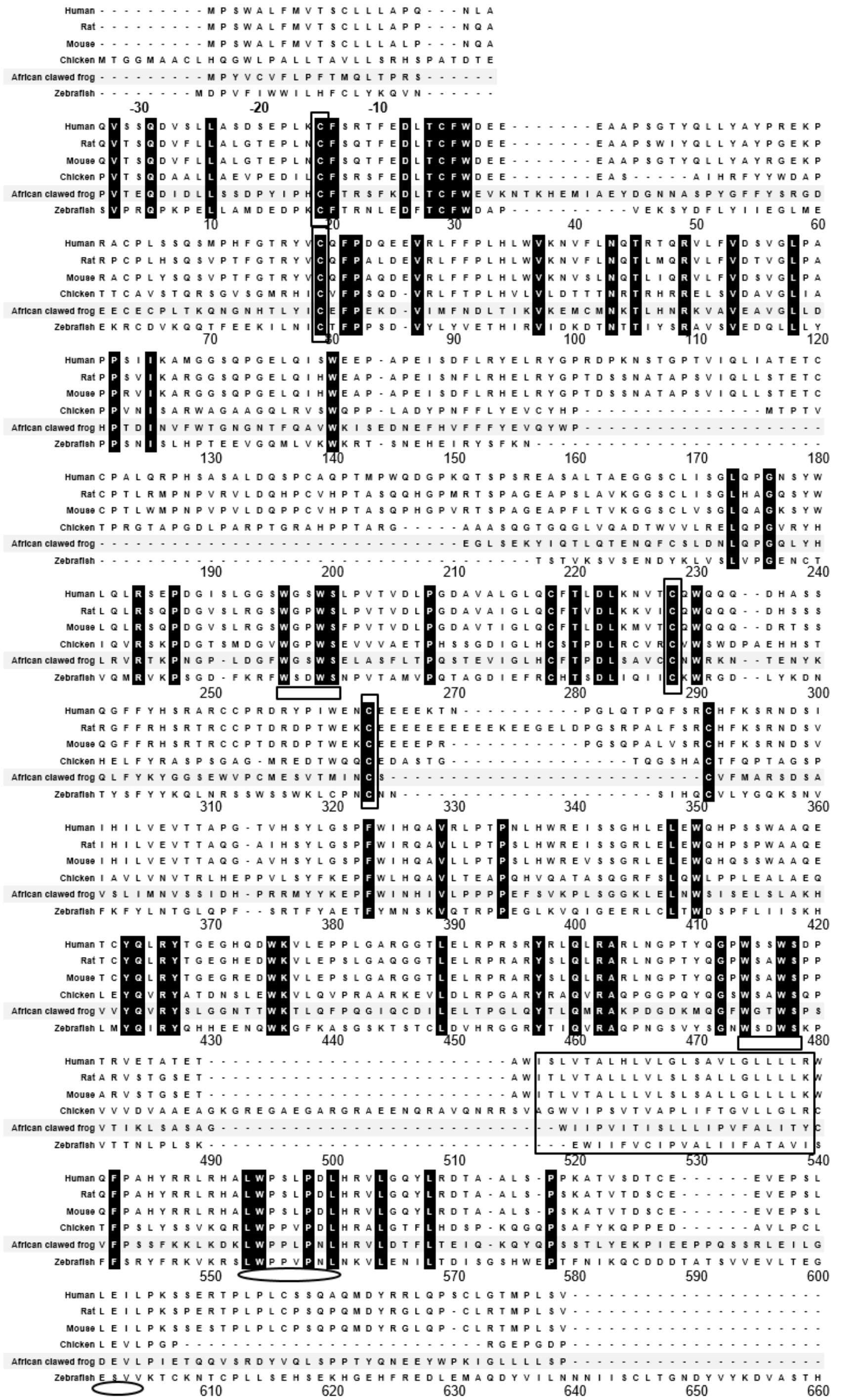

Supplementary Figure 4. Homology of Mpls from various vertebrate species. Amino acid sequence homology of mature Mpls from human, rat, mouse, chicken, zebrafish, and *X. laevis*. Numbers indicate positions from the N terminus of the mature *xl*TPO. The predicted signal peptide is shown at the top and indicated by lowercase letters. Identical amino acid residues are shaded black. Cysteine residues that form possible disulfide bridges in *xl*Mpl are boxed. Putative transmembrane domains are underlined. White boxes indicate tryptophan-serine-x-tryptophan-serine (WSXWS) motifs and the Box1 and Box2 regions, thought to be important for mitogenic signaling. Mpl sequences were obtained from GenBank (accession numbers: human, NP_005364; mouse, NP_001116421; chicken, NP_001001782; zebrafish, NP_001003858). The *xl*TPO and *xl*Mpl sequences have been submitted to DDBJ/EMBL/GenBank with accession numbers AB256538 and AB272077.

Supplementary Figure 5

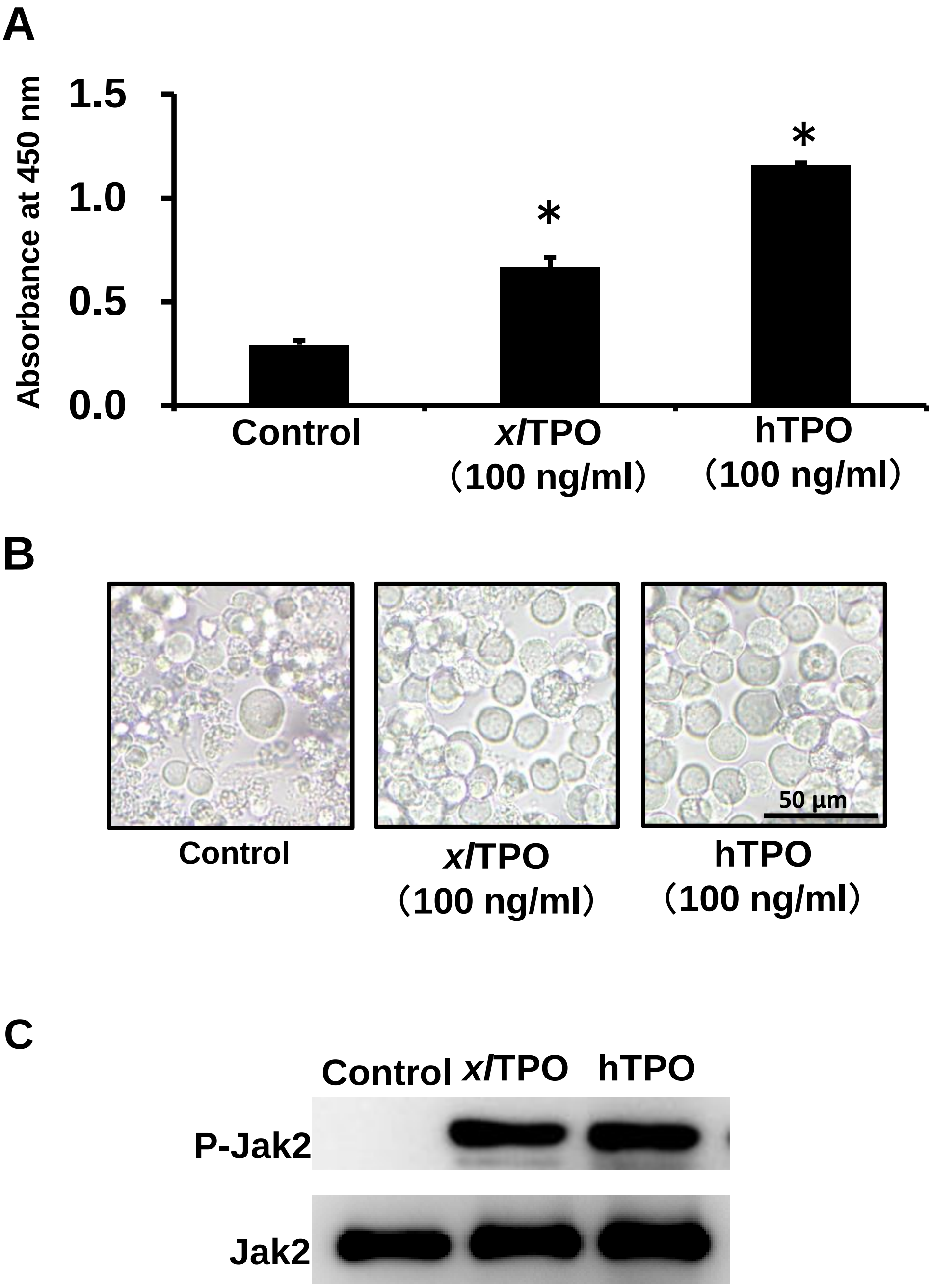

Supplementary Figure 5. Proliferation of UT-7/TPO cells after *xl*TPO stimulation. (A) MTS assay of UT-7/TPO cells. UT-7/TPO cells were stimulated with *xl*TPO or hTPO for 72 h in 96-well plates and analyzed by MTS assay. Controls were the UT-7/TPO cells cultured in the absence of TPO for 72 h. *P < 0.05 vs. the control. (B) Cell morphology of UT-7/TPO cells. UT-7/TPO cells were stimulated with *xl*TPO or hTPO for 72 h. (C) JAK2 phosphorylation. UT-7/TPO cells were cultured overnight in the absence of serum and growth factor in IMDM. Cells were stimulated with *xl*TPO or hTPO for 5 min and phosphorylated JAK2 was measured by western blotting.

Supplementary Figure 6

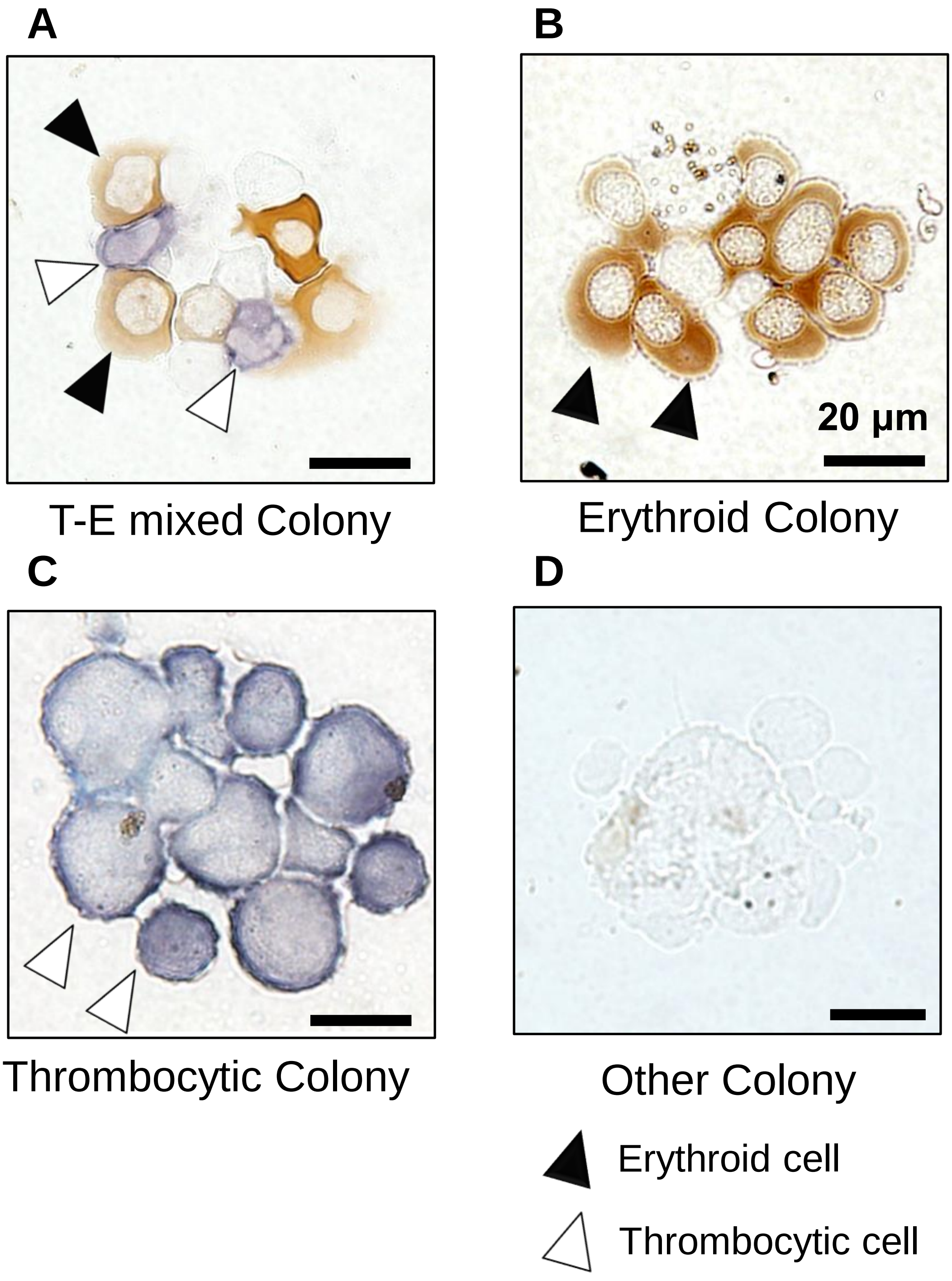

Supplementary Figure 6. Colonies derived from hepatic cells in the presence of anemic serum. Hepatic cells from anemic frogs were cultured in semi-solid medium in the presence of anemic serum. After four days, the cell were collected and cytocentrifuged smears were prepared using a cytospin cytocentrifuge. Colonies were immunostained for T12 and counterstained with *o*-dianisidine. (A) Colony of T12- and *o*-dianisidine positive cells. (B and C) Colony composed of only *o*-dianisidine or T12-positive cells. (D) Colony composed of non-hemoglobinized cells.

Supplementary Figure 7

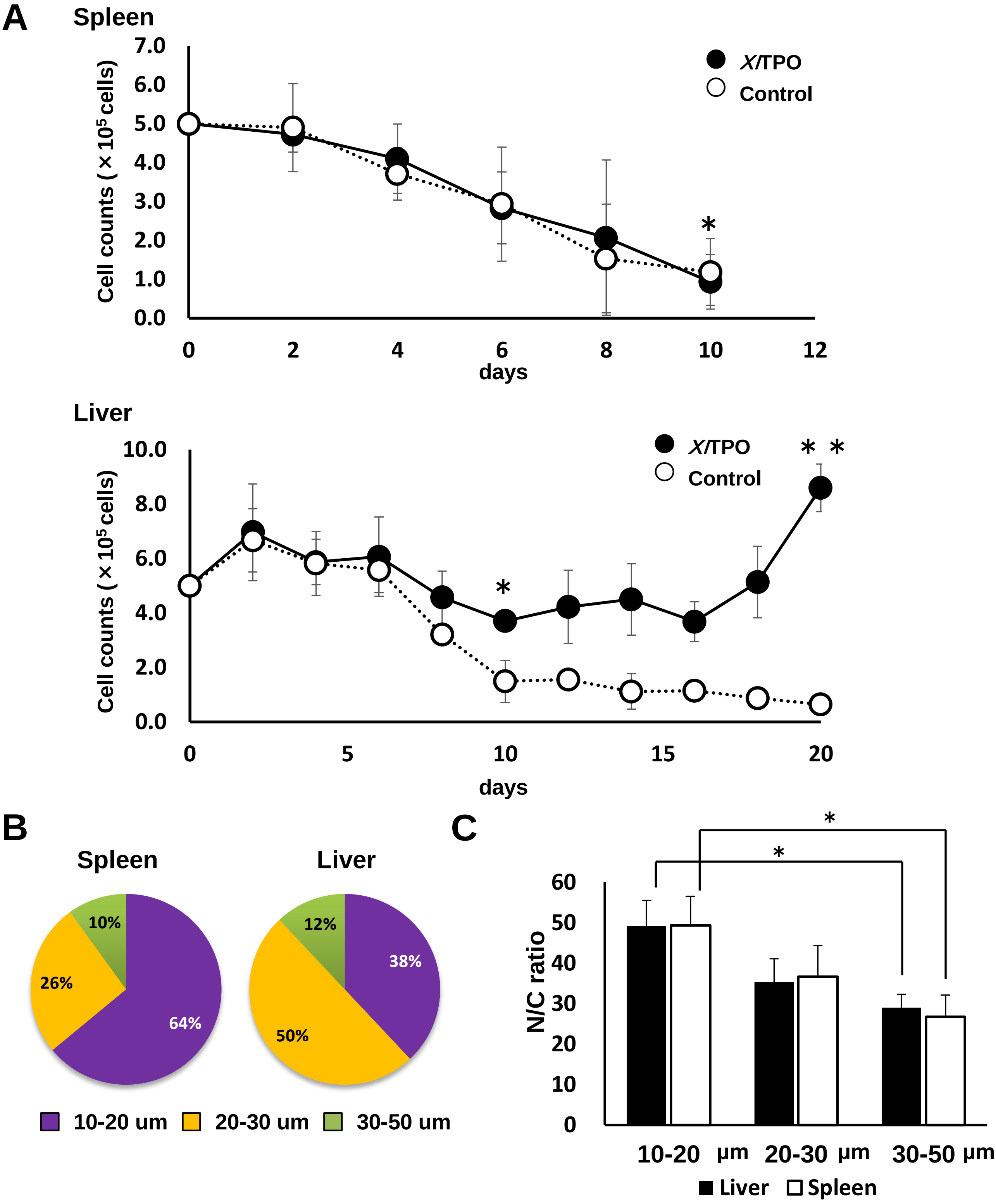

Supplementary Figure 7. Viability of splenic or hepatic cells after *x*/TPO stimulation. (A) Whole cells were counted during liquid culture of spleen or liver cells in the presence of *x*/TPO. Open squares represent the control. Black squares represent *x*/TPO-stimulated cells. *P < 0.05 vs. day 0; ** P < 0.05 vs. day 10. The data represent mean ± SD (N = 3). (B)Cellular distribution was investigated on cytospin preparations from hepatic and splenic cells after culturing with *x*/TPO for 8 days. (C) Morphological features of hepatic and splenic cells at 8 days of culture such as N/C ratio was calculated using images of cells by image-J software. Black bars indicates hepatic *x*/TPO-stimulated cells. White bars indicates splenic *x*/TPO-stimulated cells. Analysis was performed three times (N=3) . Results are presented as mean SD. *P < 0.05

Supplementary Figure 8

A

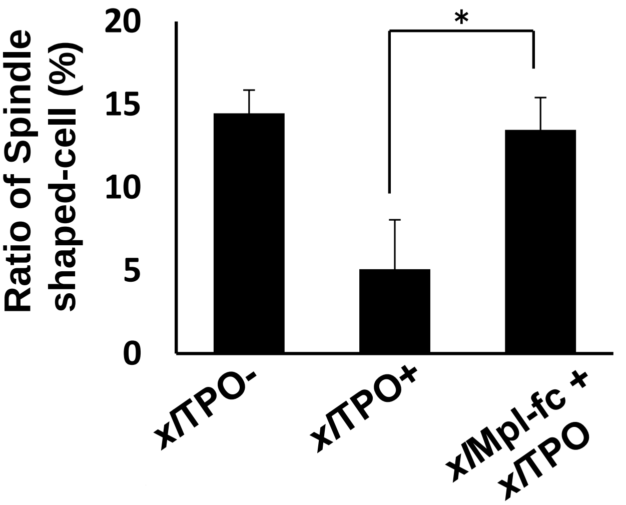

B

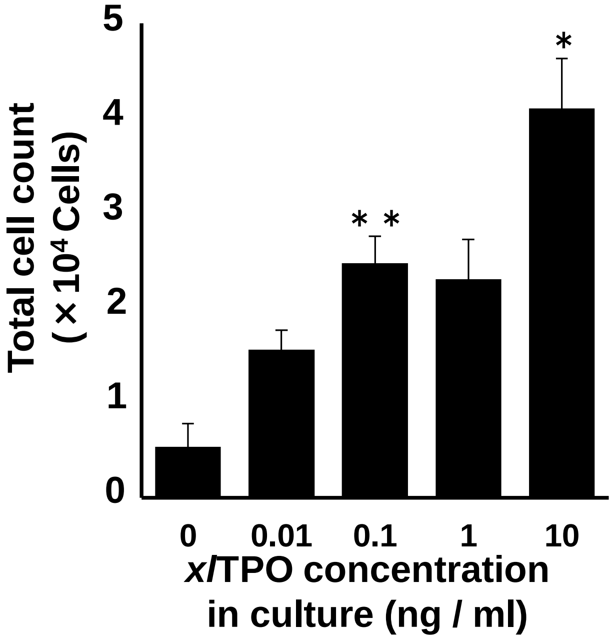

Supplementary Figure 7. *x*/TPO activity in the MKs and peripheral thrombocytes. (A) MKs were enriched by density gradient method and cultured with *x*/TPO in the presence or absence of *x*/Mpl-Fc fusion protein for 4 days. Graphs represent means + SD, n = 3. *P < 0.05 (B) Peripheral thrombocytes were enriched by density gradient method and cultured in the presence of in the presence of increasing concentrations of *x*/TPO for 8 days. Analysis was performed three times (N=3) . Results are presented as mean SD. *P < 0.05 vs 0.1 ng/mL *x*/TPO ; ** P < 0.05 vs 0 ng/mL *x*/TPO .

Supplementary Figure 9

A Full uncropped gel image from Fig. 1E

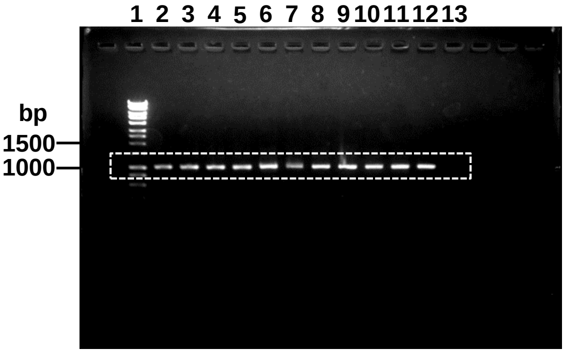

GAPDH

1: Marker	7 :Liver
2: Brain	8 :Spleen
3: Lung	9 :Kidney
4: Muscle	10:Stomach
5: Heart	11:Intestine
6:PB	12:Testis
	13:Water

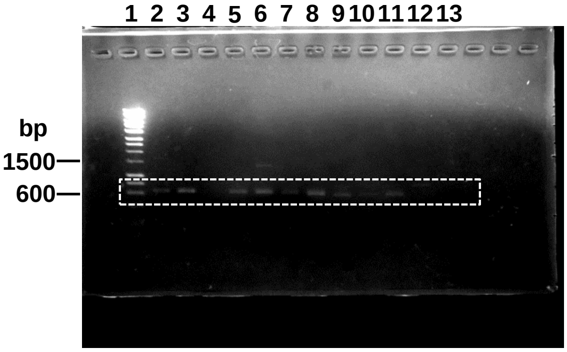

Mpl

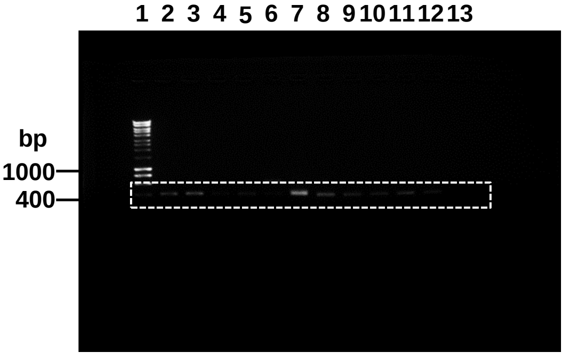

TPO

B Full uncropped gel image from Fig. 6D

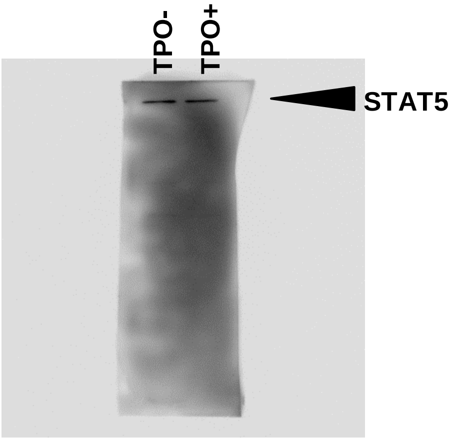

STAT5

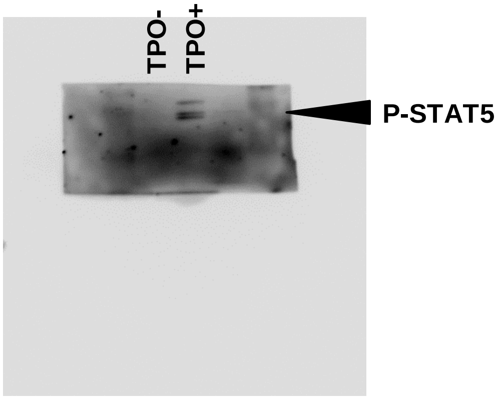

P-STAT5
